# Interpretable CNN for ischemic stroke subtype classification with active model adaptation

**DOI:** 10.1186/s12911-021-01721-5

**Published:** 2022-01-05

**Authors:** Shuo Zhang, Jing Wang, Lulu Pei, Kai Liu, Yuan Gao, Hui Fang, Rui Zhang, Lu Zhao, Shilei Sun, Jun Wu, Bo Song, Honghua Dai, Runzhi Li, Yuming Xu

**Affiliations:** 1 School of Information Engineering, Zhengzhou University, Zhengzhou, China; 2 Cooperative Innovation Center of Internet Healthcare, Zhengzhou University, Zhengzhou, China; 3 Institute of Intelligent Systems, Deakin University, Burwood, Australia; 4 The Department of Neurology, The First Affiliated Hospital of Zhengzhou University, Zhengzhou, China

**Keywords:** Interpretability, Ischemic Stroke, Active learning, Classification algorithm, Loss function

## Abstract

**Background:**

TOAST subtype classification is important for diagnosis and research of ischemic stroke. Limited by experience of neurologist and time-consuming manual adjudication, it is a big challenge to finish TOAST classification effectively. We propose a novel active deep learning architecture to classify TOAST.

**Methods:**

To simulate the diagnosis process of neurologists, we drop the valueless features by XGB algorithm and rank the remaining ones. Utilizing active learning framework, we propose a novel causal CNN, in which it combines with a mixed active selection criterion to optimize the uncertainty of samples adaptively. Meanwhile, KL-focal loss derived from the enhancement of Focal loss by KL regularization is introduced to accelerate the iterative fine-tuning of the model.

**Results:**

To evaluate the proposed method, we construct a dataset which consists of totally 2310 patients. In a series of sequential experiments, we verify the effectiveness of each contribution by different evaluation metrics. Experimental results show that the proposed method achieves competitive results on each evaluation metric. In this task, the improvement of AUC is the most obvious, reaching 77.4.

**Conclusions:**

We construct a backbone causal CNN to simulate the neurologist process of that could enhance the internal interpretability. The research on clinical data also indicates the potential application value of this model in stroke medicine. Future work we would consider various data types and more comprehensive patient types to achieve fully automated subtype classification.

**Supplementary Information:**

The online version contains supplementary material available at 10.1186/s12911-021-01721-5.

## Backgroud

Stroke is one of the leading causes of death and disability. The burden of stroke is rapidly increasing worldwide [1, 2]. As the most common type of stroke in China, Ischemic stroke (IS) patients constitute about 60%-80% in all stroke patients [3]. Therefore, it is of great significance to understand the etiological mechanism of IS for individualized treatment, prediction, prognosis and secondary prevention [4, 5]. Numerous medical studies focused on the subtype of IS [6–8]. One of the most important researches is TOAST classification, which was developed in a multicenter clinical trial of heparinoid (Org 10172) in the treatment for acute ischemic stroke [9]. TOAST classifies acute ischemic stroke into 5 subtypes: Large artery atherosis (LAA), Cardiogenic embolism (CE), Small artery occlusion (SAO), Other determined cause (OC), and Undetermined Cause (UND). It has been an effective tool in predicting various outcomes of stroke, including mortality, functional recovery, length of stay, and complications [10–12].

To determine the subtypes of IS, a trained neurologist needs to consider multiple data modalities, including patient history, laboratory tests, and medical image and so on. This process is time-consuming and introduces subjective variability. Meanwhile, it is limited by the size of dataset and experience of neurologist. At present, although machine learning method becomes a popular choice for diagnosis [13], prediction [14], prognosis [15, 16] and subtype of stroke [17], it is still plagued by the lack of interpretability. This deficiency will limit its popularization and application in the medical field. Furthermore, we could tackle the workload of neurologists through active learning regime. The essence is to select the most valuable data samples in the active cycle and append them to the training set. In the training process, active cycle is an efficient method to reduce the number of worthless training samples and save the computing resource.

In this work, we propose a causal neural network model with active model adaptation to interpretably identify TOAST subtypes of IS. The *causal* padding drives the proposed network architecture to interpretively extract patient features according to doctors’ clinical process. Meanwhile, the addition of active learning strategy with Mixed uncertainty ensures the whole training cycle more efficiently.

**Fig. 1:**
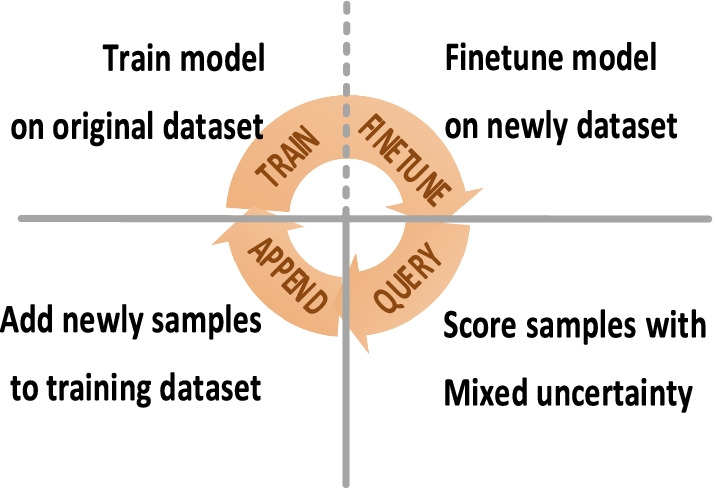
The basic schematic of our work

Figure 1 exhibits the basic schematic. It is a circulation that consists of four parts: TRAIN, FINETUNE, QUERY and APPEND. First, the original dataset is TRAINed for the initial model. QUERY calculates the selection criterion of all samples and selects the most valuable ones. APPEND adds them to the original training dataset for the FINETUNE step.

The main contributions of this work are summarized as follows:

- Utilizing active learning framework, we propose a novel causal convolutional neural network to classify IS subtype. It simulates the diagnosis process of neurologists to further enhance model interpretability.
- We design an active selection strategy, Mixed uncertainty, that actively selects samples based on dynamic trade-off between different uncertainty strategies. It could select the most representative data by considering comprehensively.
- KL-focal loss is introduced in our causal convolution neural network, which could ensure data diversity, achieve better accuracy and avoid overfitting.

## Methods

**Fig. 2:**
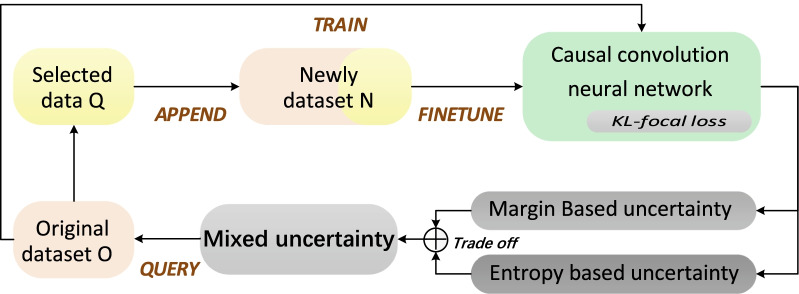
The detailed schematic of our proposed network with active model adaptation

In this work, a general framework integrating active learning and deep learning is proposed. The detailed framework could improve the interpretability of deep learning and alleviate the dilemma of insufficient medical available data, resolve the problem of overfitting and reduce the manpower consumption of data annotation in clinical application, as shown in Fig. 2. In this section, we introduce the framework in three components: Causal CNN architecture, Active selection criterion and KL-focal loss. Based on the original dataset, the customized causal convolutional neural network is adopted to train the initial model for simulating diagnosis and treatment process. The network is composed of causal convolution, which could be explained internally. Then, an active selection criterion (Mixed uncertainty) is designed to fully consider and dynamically adjust the uncertainty of samples. Actively querying the most valuable samples could reduce the onerous medical data labeling costs. Meanwhile, the size of the dataset is expanded by appending the selected samples. We use the newly dataset to finetune the initial model trained by the original dataset. Meanwhile, we equip KL-focal loss to avoid overfitting of the network and ensure the data diversity.

### Causal CNN architecture

**Fig. 3:**
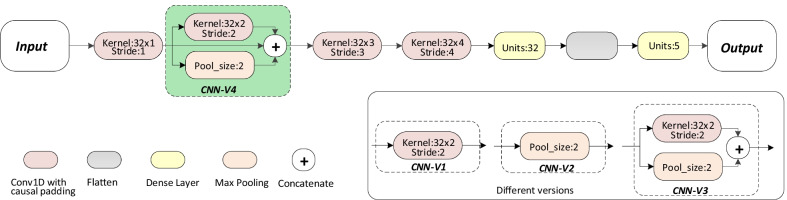
Causal CNN architecture

In this work, we design a novel Causal CNN architecture to mimic neurologists as shown in Fig. 3. The causal convolution [18] was proposed to process time series data. According to the characteristics of data, *causal* padding in the convolution is set to ensure that the model could not violate the input order. It can only operate on the input of the past time. The output of causal convolution is only related to the input of present time (*t*) and past time ($t-1$). It does not involve the information of the future time ($t+1$). In clinical practices, neurologists firstly select the most important features for diagnosis, and then secondary important features are superimposed for further diagnosis and so on. This process is sequential. We regard the final TOAST subtype as the last results given by neurologists based on all previous information. It could be described that given an input feature sequence as $x_0,x_1,x_2,...,x_t$, the output at final time *t* is $Y_t$. Meanwhile, we customize a series of convolution kernels with different strides to accelerate the convergence and enrich the receptive field. Figure 3 gives the different versions of microscopic transformations after the first causal convolution layer. CNN-V4 in green is the model proposed in this work. The implementation details are listed in the Fig. 3. The best experimental results are obtained through multi angle fusion denoted as CNN-V4. The verification is analyzed in the result section.

1 $$\begin{aligned} Y_{t}=f(x_{0}, x_{1}, x_{2},...,x_{t}) \end{aligned}$$

### Active selection criterion

The essence of active learning is to design an active data selection criterion for the training set, according to the existing training sample information, and actively select the most uncertain new samples. Based on the selected samples, the model could be further improved to make the whole process a gradual exchange process. Therefore, the key point is to establish the active selection criterion. In information theory, entropy is used to describe the uncertainty of information. Similarly, it is also used as a standard to measure the uncertainty of samples in the active selection strategy, denoted as the Entropy based uncertainty $x^a$:

2 $$\begin{aligned} x^a= arg\ max_{i=1,...,n}-\sum _{j}P(y_i\mid x_i)logP(y_i\mid x_i) \end{aligned}$$

where $y_j$ is the possibility of belonging to the *j*th category in $x_i$. $x^a$ considers the possibility of the sample belonging to each category to measure the uncertainty. When the sample is divided into all categories of possibilities with the same probability, the entropy value is the highest, that is, the sample is considered to have the greatest uncertainty. In the study of multi classification, each sample has a scoring value for the possibility of each category. The difference between the top 1 and top 2 prediction category is selected as the selection criterion $x^b$, which is based on Margin:

3 $$\begin{aligned} x^b= arg\ min_{i=1,...,n}(P(y_1\mid x_i)-P(y_2\mid x_i)) \end{aligned}$$

where $y_1$ and $y_2$ are the top 2 categories with the highest probability of $x_i$ respectively. The minimum probability difference between them means that the classifier is the least able to distinguish the specific category of the sample. The sample is considered to have the highest uncertainty. $x^b$ measures the difference between the two highest categories of possibilities, and takes the difference as uncertainty without considering the specific value. To select samples with highest uncertainty, Mixed uncertainty $x^*$ is calculated by weighting the rows of $x^a$ and $x^b$:

4 $$\begin{aligned} \begin{aligned} x^*&=arg\ min_{i=1,...,n}(\alpha *x^a+(1-\alpha )*x^b)\\&=arg\ min_{i=1,...,n}(\alpha (P(y_1\mid x_i)logP(y_1\mid x_i))\\&\quad +(1-\alpha )(P(y_1\mid x_i)-P(y_2\mid x_i))) \end{aligned} \end{aligned}$$

where $\alpha$ is a trade-off parameter to dynamically balance the two parts of Mixed uncertainty.

Benefit from the active selection criterion of Mixed uncertainty, we add selected samples into the original training dataset to finetune the initial model. This operation could not only expand the dataset, but also select more valuable samples.

### KL-focal loss

In the dataset, the distribution of TOAST subtypes is shown in Table 2. The largest number of patients with LAA is 1290, and the least number of patients with OC is 81. According to the 3 subtypes (LAA, CE and SAO) clearly defined in TOAST, the patients numbers are 1290, 107 and 550, respectively, and there is still imbalance. Therefore, we take the focal loss as the basis of loss function. Meanwhile, we use the newly dataset containing the selected samples to finetune the model trained by the original dataset, so that overfitting is easy to occur in the cyclic active learning. To overcome these limitations, we choose KL divergence developed from information theory as a regularization technique to upgrade the focal loss function. KL divergence could also be used as an indicator of data diversity. It can consolidate the diversity of data and avoid overfitting in the process of model iteration. The specific calculation of KL focal loss is as follows:

5 $$\begin{aligned} FL(p_x)= & {} -{(1-p_x)^\gamma }log(p_x) \end{aligned}$$

6 $$\begin{aligned} KL\left( P\parallel Q \right)= & {} P(x)*\left[ log(\frac{P(x)}{Q(x)})\right] \end{aligned}$$

7 $$\begin{aligned} KFL= & {} FL(p_x)+KL(P\parallel Q) \end{aligned}$$

where $\gamma$ adjusts the rate of the weight decrease. $p_x$ is the prediction result. *P*(*x*) is the estimated probability distribution of sample *x*, *Q* is the real probability distribution. The focal loss function is described in [19]. *KL*(*P*||*Q*) is the divergence between *Q* and *P*. Obviously, the smaller the divergence, the closer the estimated probability distribution is to the true distribution.

## Result

### Clinical dataset description

This dataset was collected from all the patients admitted to the department of neurology during 2014 to 2016 in a AAA Hospital. It includes 2310 stroke patients and each patient with 122 items of features. Table 1 shows Inclusion and Exclusion criteria. Professional neurologists label the TOAST subtypes for each anonymous patient records. Table 2 displays the distribution of TOAST subtypes.

**Table 1:** Inclusion and exclusion criteria

Inclusion criteria	Exclusion criteria
Age of patient over 18 years	Hemorrhagic stroke
Cerebral infarction and TIA	
Time of onset and admission over 7days	Non-cerebrovascular disease event
Sign informed consent

**Table 2:** Distribution of TOAST subtype in the cohort of patients

Etiologic subtypes of ischemic stroke	Number of patients	Proportion of subtypes (%)
Large artery atherosclerosis (LAA)	1290	56
Cardioembolism (CE)	107	5
Small artery occlusion (SAO)	550	24
Other determined cause(OC)	81	3
Undetermined cause(UND)	282	12

### Data preprocessing

We employ XGBoost to select and rank the original features in preprocessing to mimic diagnosis and treatment process of neurologists. XGBoost is a tree structure model, which could not only complete the feature selection and ranking, but also ensure the interpretability of the whole selection process. Meanwhile, the dataset is from clinic and completed by neurologists one-to-one statistics. Therefore, we choose to drop the features with feature importance $\le$ 0.005 and rank the left 93 features. Table 3 summarizes the features. Detailed feature statistics are sorted as Additional file 1.

The missing data is due to the accidental operation of the registrant, and the average integrity is 99.53%. We adopt the *mode* method to fill in the missing data without any scaling of feature values. Meanwhile, the data filling operation has also been confirmed by clinicians. It should be noted that, the operation is applied to all data sets, including training set and test set.

**Table 3:** Features of the analyzed cohort

Feature	Description
Gender	Male: 1557, Female: 753
Age	Mean age: 59.2
Demography	Nationality, Marital status, Living condition, Education level
Personal situation	Smoking, Drinking
Past medication	Antiplatelet, Antihypertensive, Antidiabetic, Antilipemic
Family history	Hypertension, Diabetes, Stroke, Cardiovascular disease
Past history	Hypertension, Stroke, TIA, Coronary atherosclerotic cardiopathy, Atrial fibrillation, Diabetes, Dyslipidemia, Renal disease, Surgery, mRS score
Treatment during hospitalization	Medication, Surgery, Rehabilitation training
Admission examination	Initial symptoms, Thrombolytic status, Basic information, NIHSS score
In-hospital adverse events	Adverse cardiac events, Adverse vascular events

### Experiments setup

In this study, all experiments are implemented based on an Intel CoreTM i7-8700K Processor at 3.70 GHz with 32 GB of RAM, one NVIDIA GeForce GTX 1080 Ti and ubuntu 18.04 operating system. We train models in Scikit-learn 0.22.1 [20] and Keras 2.2.4 with Tensrflow 1.12.0 as the backend. We adopt 10 fold cross validation to evaluate these models and epochs and batch size are set to 100 and 32.

Firstly, we construct an experiment of the comparison of 4 versions for our model to verify the most effective one.

Then we set up a set of experiments to verify the validity of the data preprocessing operations.

Next, we build a series of experiments, including machine learning and deep learning algorithms, as the baseline. Most of them are analyzed in these related work [21–24]. Meanwhile, we compare some related and advanced deep learning algorithms [25]. We select the default parameter in Scikit-learn and Keras for most models with Adam. In the LSTM-based models, the lstm-dim is set as 25.

Then we compare 8 loss functions in our task to verify the effectiveness of KL-focal loss and further extend it to other deep learning models.

Finally, we explore different strategies for active selection criterion in this task and verify the performance in individual classes.

### Evaluation metrics

The performance evaluation indicators are given by following formulas:

8 $$\begin{aligned} Accuracy= & {} \frac{TP+TN}{TP+FP+TN+FN} \end{aligned}$$

9 $$\begin{aligned} AUC= & {} Area \quad under\quad the\quad ROC\quad curve \end{aligned}$$

10 $$\begin{aligned} Precision= & {} \frac{TP}{TP+FP} \end{aligned}$$

11 $$\begin{aligned} Recall= & {} \frac{TP}{TP+FN} \end{aligned}$$

12 $$\begin{aligned} F1-score= & {} \frac{2*Precesion*Recall}{Precision+Recall} \end{aligned}$$

In the formulas TP, TN, FP and FN are for true positive, true negative, false positive and false negative respectively.

### Comparison of different versions for causal CNN architecture

Table 4 lists the performance of the causal CNN architecture at various stages of evolution to further explain the effectiveness of model customization. Figure 3 displays the architecture of causal CNN architecture and the different versions of the custom part. We left the rest of the architecture unchanged, and only the parts highlighted in green were updated for different versions. All the 4 models are based on the causal convolution to simulate the diagnosis and treatment of neurologists. The CNN-V1 achieved an accuracy of 0.5578, an AUC of 0.6557, a recall of 0.5578, a precision of 0.6012 and an F1-score of 0.4948. The CNN-V2 achieves a precision of 0.5912 and a F1-score of 0.4683, which is significantly lower than all the other models. The number of model parameters is 8997. The unique difference between CNN-V1 and CNN-V2 is the max pooling operation and the causal convolution operation in the second layer. In CNN-V3, the outputs of max pooling layer and convolution layer are spliced together with the concatenate layer. It achieves higher precision of 0.6081 and higher F1-score of 0.4973 than CNN-V1 and CNN-V2. The best classifier is CNN-V4, although the number of parameters is as high as 12,997. On the basis of CNN-V3, we continue to fuse the original output of the first causal convolution layer to derive CNN-V4. This operation changes the parameters slightly by increasing 1280 model parameters. However, it performs an accuracy of 0.6020, an AUC of 0.6757, a recall of 0.6020, a precision of 0.6213 and an F1-socre of 0.5141. Considering the improvement of performance, we choose CNN-V4 as skeleton in the following analysis.

**Table 4:** Comparison of different versions for causal CNN architecture

Model	Accuracy	AUC	Recall	Precision	F1-score	Number of parameters
CNN-V1	0.5578	0.6557	0.5578	0.6012	0.4948	11077
CNN-V2	0.5682	0.6505	0.5682	0.5912	0.4683	8997
CNN-V3	0.5652	0.6474	0.5652	0.6081	0.4973	11717
**CNN-V4**	**0.6020**	**0.6757**	**0.6020**	**0.6213**	**0.5141**	** 12997**

The bold values are to highlight our results

### Validation of the data preprocessing operations

**Table 5:** Validation of data preprocessing operations

Dataset	Accuracy	AUC	Recall	Precision	F1-score
122 raw features	0.5704	0.6484	0.5704	0.5942	0.4926
93 unranked features	0.5682	0.6479	0.5682	0.6018	0.4948
**93 ranked features**	**0.6020**	**0.6757**	**0.6020**	**0.6213**	**0.5141**

The bold values are to highlight our results

**Table 6:** Comparison of different preprocessing method

Preprocessing method	Accuracy	AUC	Recall	Precision	F1-score
Scale	0.4621	0.5927	0.4621	0.6040	0.4614
Standard Scaler	0.4534	0.5878	0.4534	0.6007	0.4541
Min–Max	0.4903	0.6052	0.4903	0.5980	0.4760
Max Abs Scaler	0.5110	0.6165	0.5110	0.6087	0.4894
L1	0.5539	0.6192	0.5539	0.5720	0.4398
L2	0.5535	0.6372	0.5535	0.5829	0.4702
**Ours**	**0.6020**	**0.6757**	**0.6020**	**0.6213**	**0.5141**

The bold values are to highlight our results

Table 5 displays the validation of data preprocessing operations in our work. It can be seen that after feature dropping with feature importance and ranking, the performance of the model changes from an accuracy and a recall of 0.5704–0.6020, an AUC of 0.6484–0.6757, a precision of 0.5942–0.6213, a F1-score of 0.4926–0.5141. Table 6 lists the comparison of different preprocessing method. **Ours** denotes that the data set we trained without any scaling of feature values. The preprocessing methods reduces the model performance by scaling the feature values.

**Fig. 4:**
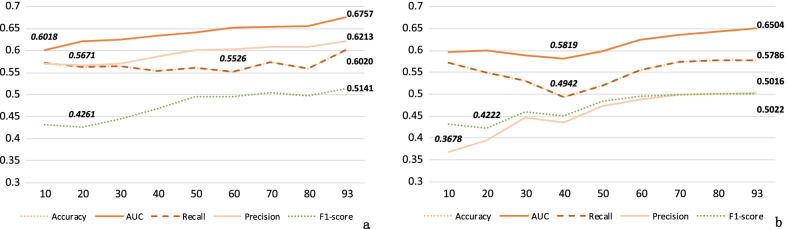
Comparison of different subsets of features with Ours and ET

Figure 4 shows the comparison of different subsets of features with **Ours** and **ET**. We set 10 as the sampling step to construct the feature subsets and select the top 10, 20...70, 80 features. We mark the highest and lowest values of the evaluation metrics. Figure 4a is the performance of different subsets of features with **Ours**. It appears that **93 features **achieves the best result in all evaluation metrics. We select **ET** as the representative of the baseline models to analyze the performance changes of different feature subsets in Fig. 4b. Although the performance does not change significantly with the number of features from 70 to 93, it still shows an upward trend.

### Comparison of different models for baseline

**Table 7:** Comparison of different models for baseline

Method	Accuracy	AUC	Recall	Precision	F1-score
NB [22]	0.5023	0.6054	0.5023	0.4493	0.4231
Multinomial NB [23]	0.1728	0.5402	0.1728	0.4471	0.2070
DT [22]	0.5421	0.6138	0.5421	0.4538	0.4594
RF [21, 22, 24]	0.5671	0.6532	0.5671	0.4865	0.4755
ET [21, 23]	0.5786	0.6504	0.5786	0.5022	0.5016
CART [24]	0.4431	0.5476	0.4431	0.4527	0.4557
GDBT [21]	0.5639	0.5956	0.5639	0.4321	0.4544
XGBoost [21]	0.5605	0.6453	0.5605	0.4734	0.4702
AdaBoost [23]	0.5409	0.5812	0.5409	0.4639	0.4716
LDA	0.5647	0.6302	0.5647	0.4577	0.4653
QDA	0.2616	0.5667	0.2616	0.4144	0.2039
LR [22, 24]	0.5565	0.6309	0.5565	0.4452	0.4290
KNN [21, 22, 24]	0.5366	0.6031	0.5366	0.4513	0.4564
SVM [21, 22, 24]	0.5646	0.6228	0.5646	0.4461	0.4570
NN [22, 26]	0.5539	0.5192	0.5539	0.3649	0.4083
MLP [23]	0.5353	0.5015	0.5353	0.3140	0.3956
LSTM	0.1295	0.5544	0.1295	0.4978	0.1252
LSTM+Att	0.0879	0.5781	0.0879	0.2701	0.0634
Bi-LSTM [25]	0.1923	0.6032	0.1923	0.7009	0.1924
Bi-LSTM+Att	0.1515	0.6020	0.1515	0.6986	0.1446
**Ours**	**0.6020**	** 0.6757**	**0.6020**	**0.6213**	**0.5141**

The bold values are to highlight our results

Table 7 enumerates the baseline results of various models, including machine learning and deep learning. We choose 16 kinds of classical machine learning models and various machine learning models mentioned in references. Among them, we classify simple neural networks (NN) and multi-layer perceptron (MLP) into machine learning algorithms. Meanwhile we select 4 LSTM based deep learning models for comparison, in which the $lstm\_dim$ is 25. Here we select CNN-V4 as our baseline model without the active adaption circulation and record it as Ours. In Table 4, we describe the detailed comparison of model version. Based on the existing clinical data, machine learning methods are generally better than deep learning methods. Among them, RF achieves the best AUC of 0.6532, ET achieves the best accuracy of 0.5786, precision of 0.5022, recall of 0.5786 and F1-score of 0.5016. Most tree structured machine learning algorithms could obtain a noteworthy baseline result. Among the deep learning methods, Bi-LSTM achieves the precision of 0.7009 and Bi-LSTM+att obtains the precision of 0.6986, which are all higher than Ours. However, LSTM-based models perform poorly in accuracy, recall and F1-socre, all of which are below 0.2. Our model outperforms all the best results listed above in all indexes except precision by attaining an accuracy of 0.6020, an AUC of 0.6757, a recall of 0.6020 and a F1-score of 0.5141. The precision of 0.6213 is also better than most of these methods.

**Table 8:** Comparison of different loss function for our model

Loss function	Accuracy	AUC	Recall	Precision	F1-score
Mean absolute error	0.4647	0.5100	0.4647	0.3886	0.3115
Mean absolute percentage error	0.4933	0.5082	0.4933	0.4457	0.3383
Mean squared error	0.5189	0.5464	0.5189	0.5085	0.3908
Mean squared logarithmic error	0.5643	0.5928	0.5643	0.5895	0.4693
Categorical Cross entropy	0.5665	0.6515	0.5665	0.5940	0.4863
Kullback leibler divergence	0.5660	0.6532	0.5660	0.5939	0.4815
Focal loss	0.2287	0.6104	0.2287	0.5704	0.2379
**KL-focal loss**	**0.6020**	**0.6757**	**0.6020**	**0.6213**	**0.5141**

The bold values are to highlight our results

### Comparison of different loss function

Table 8 exhibits the detailed results of different loss functions in this task to prove the significance of the proposed loss function. The 4 loss functions based on error perform general but stable. Among them, the performance of Mean squared logarithmic error is the best (accuracy:0.5643, AUC: 0.5928, recall: 0.5643, precision: 0.5895, F1-score: 0.4693) and that of Mean absolute error is the worst (accuracy: 0.4647, AUC: 0.5100, recall: 0.4647, precision: 0.3886, F1-score: 0.3115). The recall of Focal loss is 0.2287 and the F1-score is 0.2379, which are significantly lower than the results of other loss functions. The performance of Kullback leibler divergence loss function (accuracy: 0.5660, AUC: 0.6532, recall: 0.5660, precision: 0.5939, F1-score: 0.4815) and Categorical cross entropy loss function (accuracy: 0.5665, AUC: 0.6515, recall: 0.5665, precision: 0.5940, F1-score: 0.4863) are the closest to the best performance of KL-focal loss. KL-focal loss obtains an accuracy of 0.6020, an AUC of 0.6757, a recall of 0.6020, a precision of 0.6213 and a F1-score of 0.5141. We integrate KL regularization and focal loss, and combine the advantages of them. KL regularization could trade off the distance in the iterative process, and could keep the diversity of data. Focal loss could further alleviate the limitation of imbalance.

**Fig. 5:**
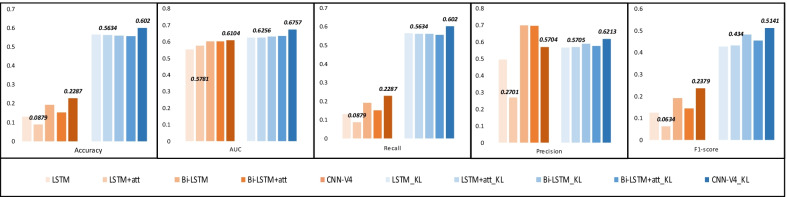
Comparison of different models for KL-focal loss. The orange ones are the result of using Focal Loss, the blue ones are the result of using KL-focal loss function

### Comparison of different models for KL-focal loss

To demonstrate the generalization ability of KL-focal loss function, we equip the loss function with LSTM-based models in Fig. 5. It appears that the loss function not only improves our model, but also has remarkable adaptability to LSTM-based models. It could greatly improve the performance of the models in terms of accuracy, recall and F1-score, except for the precision of Bi-LSTM based models. We take LSTM+att model as an example to analyze. The KL-focal loss has the most significant effect on accuracy, recall and F1-score. The accuracy and recall of LSTM+att model increases from 0.0879 to 0.5634, and the F1-score increases from 0.0634 to 0.4340. It leads to more than 5 times improvement. The precision increased by 2 times, from 0.2701 to 0.5705. Similarly, there are obvious improvements in other LSTM-based models. Although in the item of precision, Bi-LSTM-based models show a decline, the models are all improved to achieve a similar and more balanced performance.

**Fig. 6:**
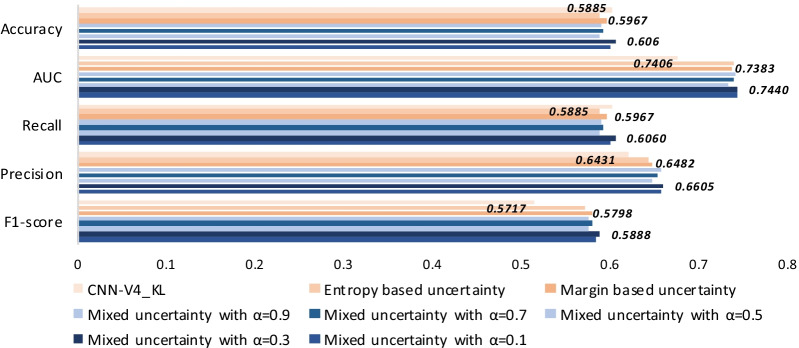
Comparison of different strategies for active selection criterion

### Comparison of different strategies for active selection criterion

Figure 6 verifies the performance of Mixed uncertainty strategy as the active selection criterion in this work. According to previous results, the comparison is conducted on the CNN-V4 model equipped with KL-focal loss. The results show that the introduction of active learning could improve the performance of our model. The Mixed uncertainty with $\alpha$ = 0.3 shows the best performance. It achieves an accuracy of 0.6060, an AUC of 0.7440, a recall of 0.6060, a precision of 0.6605 and an F1-score of 0.5888. However, the effect of the two independent active selection strategies is worse than that of the mixed ones. The Entropy based uncertainty achieves an AUC of 0.7406, a recall of 0.5885, a precision of 0.6401 and an F1-score of 0.5717. The Margin based uncertainty attains an accuracy of 0.5967, an AUC of 0.7383, a recall of 0.5967, a precision of 0.6482 and an F1-score of 0.5798. With the change of parameters $\alpha$ , the performance of Mixed uncertainty also has a slight swing. After the experimental verification, we finally choose the parameter of 0.3. It is worth noting that in the data append process, we select the top 50% of the samples, about 1200 samples. In the finetune process, the result in Fig. 6 is obtained by only once active adaption circulation.

**Fig. 7:**
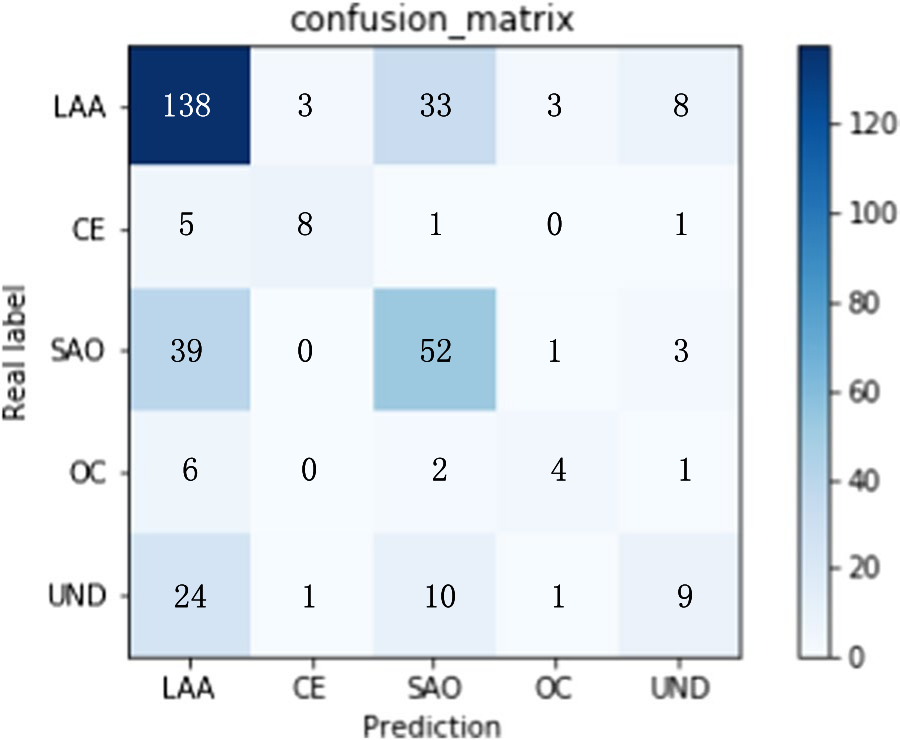
Confusion matrix of the best model

With the increase of datasets, the time complexity of the model will increase correspondingly without causing more time consumption. Due to the limitation of dataset and GPU computing power, our work does not need to worry about the burden of time consumption. The confusion matrix of the best model is displayed in Fig. 7. The detailed etiological distribution of the addition patients is shown in the Table 9. The number of SAO patients increased the most, 400, up to 72%, followed by OC patients, an increase of 60%, a total of 49. Because the number of such patients in the original data set is only 81. The largest number of is LAA patient, and its increase rate is the least, 42%. CE and UND increased by 44% and 56% respectively. Table 10 lists the comparison of classification performance in individual classes. ‘*’ indicates the model results without adding the active learning cycle. Numbers in italics in parentheses indicate the changes of model performance. ‘+’ indicates increase and ‘−’ indicates a decline in the evaluation metrics. It appears that the classification performance of SAO is improved most obviously. The precision increased from 0.3966 to 0.5392, the recall increased from 0.2821 to 0.5392, the F1-score from 0.2910 to 0.5446. There is a decrease in recall and F1-score in LAA, due to the lowest percentage 42% increase in addition patients in the active learning cycle. However, the other four diseases and their overall performance improved.

**Table 9:** Distribution of TOAST subtype in the addition patients

Etiologic subtypes of ischemic stroke	Number of patients	Proportion of initial data (%)
Large artery atherosclerosis (LAA)	545	+ 42
Cardioembolism (CE)	47	+ 44
Small artery occlusion (SAO)	400	+ 72
Other determined cause(OC)	49	+ 60
Undetermined cause(UND)	159	+ 56

**Table 10:** Comparison of classification performance in individual classes

Subtype	Precision*	Recall*	F1-score*	Precision	Recall	F1-score
LAA	0.5960	0.8774	0.7042	0.6559 (*+ 0.0599*)	0.7552 (−* 0.1222*)	0.6994 (−* 0.0048*)
CE	0.6220	0.3857	0.4705	0.7208 (*+ 0.0988*)	0.5343 (*+ 0.1486*)	0.5923 (*+ 0.1218*)
SAO	0.3966	0.2821	0.2910	0.5690 (*+ 0.1724*)	0.5392 (*+ 0.2571*)	0.5446 (*+ 0.2536*)
OC	0.2917	0.0682	0.1020	0.4785 (*+ 0.1868*)	0.2747 (*+ 0.2065*)	0.3277 (*+ 0.2257*)
UND	0.3067	0.0280	0.0507	0.3943 (*+ 0.0876*)	0.2391 (*+ 0.2111*)	0.2825 (*+ 0.2318*)

## Discussion

Although the TOAST subtype could be determined by experienced neurologists after synthesizing clinical information, it is difficult for general physicians to make a correct diagnosis. Meanwhile, the consensus among scholars on TOAST subtype is only in a moderate level. Many scholars studied the clinical application of TOAST subtype [27–31]. In addition, more studies focused on systematic reviews and meta-analysis to describe the prognosis and distribution of TOAST subtype [32, 33]. Although these clinical studies have achieved certain degree of success, additional manual work is needed to extract features to apply these research results. Recently, machine learning methods have been a powerful tool for precision medicine in stroke [17, 21–24, 26]. Meanwhile, these methods are also applied to different data formats [25, 34–36]. Nevertheless, applications of machine learning for TOAST subtypes classification is very scarce. On the one hand, controversy over the consistency of TOAST in the medical field limits this kind of research. On the other hand, the interpretability of clinical machine learning methods needs further research to improve its application. In this work, we propose a causal CNN with active model adaptation to classify TOAST subtype. We firstly select and rank features based on the importance analysis of machine learning algorithms. This process ensures interpretability from the data source. Then a customized causal CNN skeleton enhances intrinsic interpretability. Next, we design selection criterion in active model adaptation to solve the performance constraints caused by insufficient data. Furthermore, the modified KL-focal loss avoids the occurrence of overfitting in the cycle and ensures the diversity of data.

### Limitations and future considerations

Our study has several limitations. First, although our work focuses on the inherent interpretability of the model, the effect of the baseline model is indeed limited. The consistency of TOAST classification and applicability in different regions are controversial in clinic, which is reflected in the unsatisfactory classification effect of many baseline models. We will continue to focus on the localization of TOAST and further optimize the patient classification criteria.

Second, our research focuses on the design and optimization of classification model and simply removes the features whose feature importance is less than 0.005. Although we verify the selection of features, we do not consider the influence of different feature combinations. More comprehensive optimization data could provide more accurate prediction, which could not only improve the performance of the model, but also provide risk factor analysis for stroke etiology classification.

Then, although we retain many features, there are still some other data types in clinic for etiological typing prediction, including laboratory values, diagnostic tests, imaging and reports. Multiple data types could more comprehensively reflect and provide patient information that better matches TOAST.

The last limitation is that our data comes from the manual statistics of the same clinical hospital department, and the amount of data is limited, although the patient information has been counted for three years. Meanwhile, the single center data source must be further expanded to enhance the clinical value and significance of our work.

## Conclusion

With the development of precision medicine and personalized healthcare, disease subtype classification plays an increasingly important role in prediction, treatment and prognosis. Although a large number of clinical data could provide strong support for disease subtype classification, manual classification is resource intensive and time-consuming, which limits the development. Automatic subtype classification based on computer-aided technology has become a more powerful tool. This study attempts to explore an automated IS subtype classification method based on machine learning technology on clinical data. We construct a backbone causal convolutional neural network to simulate the diagnosis and treatment process of neurologists. Active learning is introduced to reduce the workload and further improves the performance with the designed Mixed uncertainty. Finally, we upgrade the focal loss function by combining with KL regularization to robustly distinguish different subtypes of IS.

Future work could extend our method to EHR documents and medical records to achieve fully automated subtype classification and focus on patients who do not meet inclusion criteria. In addition, we will leverage unsupervised method to further verify TOAST effectiveness and practicality.

## Supplementary Information

**Additional file 1: Table S1.** The 93 features in order of feature importance obtained by XGBoost.

## Data Availability

The datasets generated and/or analyzed during the current study are not publicly available due to the hospital’s regulations, but are available from the corresponding author on reasonable request.

## Abbreviations

IS: Ischemic stroke
TIA: Transient ischemic attacks
LAA: Large artery atherosis
CE: Cardiogenic embolism
SAO: Small artery occlusion
OC: Other determined cause
UND: Undetermined cause
CNN: Convolutional neural network
KL: Kullback Leibler divergence
FL: Focal loss
NB: Naïve Bayes
DT: Decision tree
RF: Random forest
ET: Extra trees
CART: Classification and regression tree
GDBT: Gradient boosting decision tree
XGBoost: EXtreme gradient boosting
AdaBoost: Adaptive boosting
LDA: Linear discriminant analysis
QDA: Quadratic discriminant analysis
LR: Logistic regression
KNN: K-nearest neighbour
SVM: Support vector machine
NN: Neural network
MLP: Multi-layer perceptron
LSTM: Long short-term memory
Att: Attention mechanism
