# Supplementary material for: Interpretable CNN for ischemic stroke subtype classification with active model adaptation
Source: BMC Med Inform Decis Mak. 2022 Jan 5;22:3. doi: 10.1186/s12911-021-01721-5 (PMC8729146; doi:10.1186/s12911-021-01721-5)
Supplement: Supplementary file 1 — Additional file 1: Table S1. The 93 features in order of feature importance obtained by XGBoost. [file 12911_2021_1721_MOESM1_ESM.docx]

**Table S1.** The 93 features in order of feature importance obtained by XGBoost

| **Feature Description (1-31)** | **Integrity** | **Feature Description (32-61)** | **Integrity** | **Feature Description (62-93)** | **Integrity** |
| --- | --- | --- | --- | --- | --- |
| Atrial fibrillation | **100%** | Place a nasogastric tube | **100%** | Education level | **98.96%** |
| Angina pectoris during hospitalization | **100%** | Antiplatelet therapy during hospitalization | **100%** | Antiplatelet therapy | **100%** |
| Myocardial infarction during hospitalization | **100%** | Hormone or immunosuppressant drugs during hospitalization | **99.57%** | Incontinence | **100%** |
| Atrial fibrillation during hospitalization | **100%** | Passive smoking | **100%** | Transient ischemic attack | **100%** |
| Surgical operations during hospitalization | **100%** | Whether to thrombolytic | **100%** | Sensory symptoms | **100%** |
| Anticoagulation treatment during hospitalization | **100%** | Decreased muscle strength | **99.83%** | Diabetes | **100%** |
| Lack of vision | **99.83%** | Family history of cardiovascular disease | **100%** | Hypertension | **100%** |
| History of subarachnoid hemorrhage 100% | **100%** | Emotional abnormalities | **100%** | Heart rate on admission | **100%** |
| Cognitive dysfunction | **99.83%** | Type of hypoglycemic drugs | **100%** | Family history of hypertension | **100%** |
| Nation | **100%** | Gastrointestinal bleeding during hospitalization | **100%** | Time from onset to admission | **100%** |
| Anticoagulant therapy | **99.91%** | First NIHSS score after admission | **100%** | Type of antihypertensive drugs | **100%** |
| Cerebral infarction during hospitalization | **100%** | History of cerebral infarction | **100%** | Cerebral hemorrhage | **100%** |
| Rehabilitation of swallowing function | **100%** | Walk ability within 48 hours after admission | **99.78%** | Diastolic blood pressure control level | **100%** |
| Double vision | **99.83%** | Marital status | **100%** | Systolic blood pressure control level | **100%** |
| mRS score before onset | **100%** | Peripheral arterial events during hospitalization | **100%** | Antithrombotic method | **99.57%** |
| Consciousness disturbance | **99.83%** | Previous operations and surgeries | **100%** | Antihypertensive treatment during hospitalization | **100%** |
| Blurred vision | **99.35%** | Hemorrhage transformation after infarction during hospitalization | **100%** | Family history of diabetes | **100%** |
| Type of lipid-regulating drugs during hospitalization | **98.70%** | Hypoglycemic treatment | **100%** | Abnormal gait | **100%** |
| Type of anticoagulant drugs during hospitalization | **100%** | Type of hypoglycemic drugs during hospitalization | **100%** | [Systolic](javascript:;) [pressure](javascript:;) | **100%** |
| Drugs for regulating blood lipids | **96.54%** | History of stroke | **100%** | Height | **100%** |
| Pulmonary infection during hospitalization | **100%** | Waist | **100%** | Diastolic pressure | **100%** |
| Fall attacks | **99.35%** | Type of antiplatelet | **100%** | The swallowing evaluation result | **100%** |
| Seizures | **99.83%** | Fasting during hospitalization | **100%** | Drinking | **99.78%** |
| Kidney disease | **100%** | Antibiotics during hospitalization | **99.57%** | Weight | **100%** |
| Lipid metabolism disorders | **100%** | Coronary heart disease 100% | **100%** | Hypertension | **100%** |
| Aphasia | **99.35%** | Swallowing function evaluation before oral ingestion, water, or medication | **97.84%** | Dysarthria | **100%** |
| Type of coronary heart disease | **100%** | Age | **100%** | Vertigo | **100%** |
| The name of the operation during hospitalization | **100%** | Family history of stroke | **100%** | Type of antiplatelet during hospitalization | **96.54%** |
| Difficulty swallowing | **99.83%** | Time of last infarction | **100%** | Gender | **100%** |
| Type of antihypertensive during hospitalization | **100%** | Living conditions | **99.13%** | History of surgery in last 3 months | **100%** |
| Headache | **99.83%** | Occur inhalation pneumonia | **100%** | Heart failure during hospitalization | **100%** |
